# Curing Rheumatism with Celery

**Published:** 1885-08

**Authors:** 


### Curing Rheumatism With Celery.

A German correspondent of an English paper writes as follows : I have had a severe attack of inflammatory rheumatism and was healed in two days' time by a soup made of the stalks and roots of celery : therefore I desire to make this simple remedy known through the columns of your paper for the benefit of all suffering from gout or rheumatism of any form. I was induced to try it by seeing the following notice: "Numerous cures of rheumatism by the use of celery have recently been announced in English papers." New discoveries—or what claim to be discoveries—of the healing virtues of plants are continually being made. One of the latest is that celery is a cure for rheumatism; indeed, it is asserted the disease is impossible if the vegetable is cooked and freely eaten. The fact that it is always put on the table raw prevents its therapeutic powers from being known. The celery should be cut into bits, boiled in water until soft, and the water drank by the patient. Serve warm with pieces of toasted bread, and the painful ailment will soon yield. Such is the declaration of a physician who has again and again tried the experiment, and with uniform success. At least two-thirds of the cases named "heart disease" are ascribed to rheumatism and its agonizing ally, gout. Small pox, so much dreaded, is not half so destructive as rheumatism, which, it is maintained by many physicians, can be prevented by obeying nature's laws in diet. Here, in Germany, we boil the root and stalks, as the root is the principal part of it, and afterward eat it as a salad with oil and vinegar. I received such immediate benefit that I am anxious to let all the rheumatic sufferers know of it.

